# Calprotectin and cross-linked N-telopeptides of type I collagen levels in crevicular fluid from implant sites with peri-implant diseases: a pilot study

**DOI:** 10.1186/s40729-018-0138-2

**Published:** 2018-09-13

**Authors:** Eijiro Sakamoto, Rie Kido, Yoritoki Tomotake, Yoshihito Naitou, Yuichi Ishida, Jun-ichi Kido

**Affiliations:** 1 Department of Periodontology and Endodontology, Institute of Biomedical Sciences, Tokushima University Graduate School, 3-18-15 Kuramoto, Tokushima, 770-8504 Japan; 2 Oral Implant Center, Tokushima University Hospital, Tokushima, Japan; 3 Department of Oral and Maxillofacial Prosthodontics, Institute of Biomedical Sciences, Tokushima University Graduate School, Tokushima, Japan

**Keywords:** Calprotectin, NTx, Peri-implant crevicular fluid, Peri-implant diseases

## Abstract

**Background:**

Peri-implant crevicular fluid (PICF) contains calprotectin and NTx, which are markers for inflammation and bone resorption, respectively. The aims of this pilot study were to compare calprotectin and NTx levels in PICF from implant sites with or without peri-implant diseases and to evaluate the usefulness of calprotectin and NTx as diagnostic markers for peri-implant diseases.

**Methods:**

Thirty-five patients with dental implants participated in this pilot study. PICF samples were collected from peri-implant disease sites (*n* = 40) and non-diseased (healthy) sites (*n* = 34) after clinical indicators including probing depth (PD), bleeding on probing (BOP), gingival index (GI), and bone loss (BL) rate were investigated. Calprotectin and NTx amounts in PICF were measured using their respective ELISA kits and then compared between diseased and healthy samples. The relationship between PICF calprotectin or NTx levels and clinical indicator levels was investigated. A receiver operating characteristic (ROC) curve analysis of calprotectin and NTx was performed to predict peri-implant diseases.

**Results:**

Calprotectin and NTx levels in PICF were significantly higher from peri-implant disease sites than from healthy sites. PICF calprotectin amounts correlated with PD, and its levels were significantly higher in the GI-1 and GI-2 groups than in the GI-0 group. PICF NTx amounts correlated with PD and the BL rate. ROC curves indicated that PICF calprotectin and NTx are useful biomarkers for peri-implant diseases.

**Conclusions:**

Calprotectin and NTx in PICF have potential as biomarkers for the diagnosis of peri-implant diseases.

## Background

Dental treatments with implants are now being widely performed due to advances in the development of surgical procedures for dental implants and prosthodontics. However, the incidence of peri-implant diseases has been increasing with implant placement [1], and thus, the early detection of these diseases is important for maintaining dental implants. Peri-implant diseases with inflammation and the destruction of peri-implant tissues have mainly been classified into peri-implantitis with the resorption of alveolar bone around osseointegrated dental implants and peri-implant mucositis without pathological bone resorption [2]. Peri-implant diseases are diagnosed by clinical indicators including probing depth (PD), bleeding on probing (BOP), suppuration, the mobility of an implant, and radiographic bone loss (BL) [3, 4]. Clinical indicators for a diagnosis of peri-implant diseases are similar to the diagnostic indicators for periodontal diseases of natural teeth. However, the measurement of PD using a dental probe is more difficult around dental implants than around natural teeth because peri-implant tissues have less attached gingiva compared with periodontal tissue, and implant structures and prosthetic superstructures sometimes prevent a probing [3, 5]. BL of 2–3 mm on radiographs has been used as a diagnostic standard in cumulative interceptive supportive therapy (CIST) [6]; however, difficulties are associated with obtaining accurate information on slight BL on radiographs in conventional X-ray examinations. The prevalence of peri-implant mucositis and peri-implantitis was previously reported to be between 19 and 65% and between 1 and 47%, respectively [1, 7], and showed a wide range because case definition of peri-implant diseases was different among those studies in which peri-implant diseases were diagnosed using clinical indicators. These reports suggest that the case definition with the diagnosis of peri-implant diseases using clinical indicators is not sufficiently accurate or clear to evaluate pathological conditions.

The diagnosis of peri-implant diseases using biomarkers in peri-implant crevicular fluid (PICF) has recently been examined and may be more accurate than that of clinical indicators to evaluate inflammation and the degradation of tissue surrounding dental implants [4, 7, 8]. PICF contains similar components to gingival crevicular fluid (GCF), namely pro-inflammatory cytokines such as interleukin-1β (IL-1β) and tumor necrosis factor-α (TNF-α), enzymes including aspartate aminotransferase (AST) and collagenase-2 (matrix metalloproteinase-8 (MMP-8)), and bone-related proteins such as cross-linked C-telopeptide of type I collagen (ICTP) and receptor activator of nuclear factor-κB (NF-κB) ligand (RANKL) [9–13]. These factors and proteins in PICF and GCF are regarded as diagnostic biomarkers for peri-implant diseases as well as periodontal diseases.

Calprotectin (S100A8/S100A9) is an inflammation-related protein that is produced in leukocytes, macrophages/monocytes, and epithelial cells, and its level increases in several inflammatory diseases including ulcerative colitis, rheumatoid arthritis, and cystic fibrosis [14, 15]. Calprotectin was previously detected in GCF, and its level was significantly higher in GCF from periodontal disease sites than in that from healthy non-diseased sites [16, 17]. Furthermore, GCF calprotectin levels correlated with clinical indicator levels, such as PD, GI, and BOP [17, 18], and was shown to predict periodontal disease activity [19]. These findings indicate that calprotectin is a useful inflammatory biomarker for periodontal diseases. Calprotectin was also detected in PICF, but its levels in PICF samples from healthy and peri-implant disease sites were not compared [20].

Cross-linked N-telopeptide of type I collagen (NTx) is a product of bone type I collagen degradation by cathepsin K in osteoclasts, is released into blood and urine, and is a specific biomarker of bone resorption [21–23]. NTx levels have been shown to increase in the blood and urine of patients with osteoporosis, hyperparathyroidism, and bone metastasis of cancer and are used as a diagnostic marker for these bone metabolism diseases [23, 24]. GCF contains NTx, and significant differences were not detected in its levels in GCF between healthy and periodontitis sites [25–27]. In contrast, Aruna [28] examined NTx in GCF samples from periodontitis sites and did not detect NTx in GCF from healthy sites. Although Friedmann et al. [20] measured NTx amounts in PICF and GCF, its levels in PICF did not correlated with changes of alveolar bone levels.

This pilot study aims to investigate whether calprotectin and NTx levels in PICF reflect inflammation and alveolar BL in peri-implant tissues, respectively, and also if these proteins are useful biomarkers for the diagnosis of peri-implant diseases.

## Methods

### Patients and clinical examinations

The present clinical study was approved by the Ethics Committees of Tokushima University Hospital (nos. 2368 and 2719) in accordance with the Helsinki Declaration of 2013 and performed from November 2016 to August 2017. Patients who received dental implants from 3 to 9 years ago, had healthy or diseased implants with peri-implant diseases, and visited at Tokushima University Hospital for the maintenance of dental implants and treatment were recruited for the present clinical study. Thirty-five patients (10 males and 25 females; aged 68.7 ± 6.5 years) gave written informed consent after receiving an explanation of this study (Table 1). Participants with healthy and diseased dental implants did not have any systemic inflammatory diseases or a history of antibiotic therapy within 3 months. PD, BOP, and gingival index (GI) were examined as clinical indicators after the collection of PICF. GI scores were evaluated according to modifications of the standard of Löe and Silness [29]. The BL rate of alveolar bone was assessed on radiographic films according to modifications of Schei et al.’s method [30]. Diseased sites with peri-implant diseases were defined as periodontal sites with PD ≥ 3 mm, BOP negative or positive, and GI score ≥ 1. Healthy implant sites were defined as sites with PD < 3 mm, BOP negative, and GI score = 0.

**Table 1:** Characteristics of participants and examining sites

Participants		
Number of participants	35	
Gender (male/female)	10:25	
Age (years)	68.7 ± 6.5	
Examining sites	Healthy	Diseased
Number of PICF samples	34	40
PD (mm)	2.32 ± 0.58	4.70 ± 1.36^†^
Gingival index	0.0 ± 0.0	1.5 ± 0.5^†^
BOP-positive rate (%)	0.0 ± 0.0	40.0 ± 15.2*
Bone loss rate (%)	19.7 ± 9.8	42.7 ± 18.0^†^

*P < 0.01 and ^†^*P* < 0.001 vs healthy group

### PICF sampling and sample preparation

PICF samples were collected from peri-implant sites using sterile paper strips according to a modified procedure of our previous method [31]. Briefly, PICF sampling sites were isolated with cotton rolls, supra-gingival plaque was removed, and sites were then very gently air-dried. Periopaper® (Oraflow Inc., NY, USA) was gently inserted into a peri-implant crevice and held for 30 s. The volume of PICF was measured using a Periotron® 8000 (Harco Electronics, Winnipeg, MB, Canada). Paper strips containing blood and pus were not used in the present study. PICF in the paper strip was extracted in 100 μl of phosphate-buffered saline (pH = 7.4) containing 0.2 μM phenylmethylsulfonyl fluoride by centrifugation and used in ELISA for calprotectin and NTx.

### Protein determination by ELISA

Calprotectin in PICF samples was determined using Calprotectin Human ELISA kit® (Hycult Biotech, PB Uden, the Netherlands) according to the instruction manual. Briefly, the extracted PICF solution was diluted to 100–200-fold using dilution buffer provided in the kit. The diluted PICF solution was added to wells coated with an antibody of human calprotectin and incubated at room temperature for 1 h. After washing the wells, a biotinylated anti-calprotectin antibody was added and incubated at room temperature for 1 h. An immune complex in the wells was reacted with a streptavidin-peroxidase conjugate for 1 h and further incubated with 3,3′,5,5′-tetramethylbenzidine (TMB) for 15 min in the dark. After stopping the reaction using a stop solution, the absorbance of the reacting solution in wells was determined using a microplate reader at 450 nm.

NTx in PICF samples was measured using Human NTx-I ELISA kit® (LifeSpan Biosciences Inc., Seattle, WA, USA) according to the instruction manual. Briefly, extracted PICF samples were added to wells and incubated at 37 °C for 90 min. A biotinylated anti-NTx antibody was added to the wells containing PICF sample solution and incubated at 37 °C for 1 h with gentle agitation. After washing the wells, HRP conjugate was added, incubated at 37 °C for 30 min, and then reacted with TMB substrate solution at 37 °C for 15 min. After stopping the reaction, the absorbance of the reacting solution was determined using at 450 nm. The concentrations of calprotectin and NTx were expressed as nanograms per microliter of PICF.

### Statistical analysis

Differences in PD, GI, the BL rate, calprotectin levels, and NTx levels between healthy and diseased groups were statistically analyzed by the Mann-Whitney *U* test. Differences in the BOP-positive rate between healthy and diseased groups were statistically evaluated using Fisher’s exact test. Difference in calprotectin amounts among the GI score 0, 1, and 2 groups were analyzed by the Mann-Whitney *U* test. The relationships between PD and calprotectin or NTx amounts and between the BL rate and NTx amount were analyzed by Spearman’s rank correlation test. Receiver operating characteristic (ROC) curves was constructed for calprotectin and NTx amounts in the healthy and diseased groups. Data were analyzed using statistical analysis software (SPSS version 20, IBM, Chicago, IL, USA). *P* values less than 0.05 were considered to indicate significance.

## Results

### Characteristics of PICF samples and sites of PICF collection

Thirty-four of PICF samples were collected from healthy peri-implant sites and forty samples from diseased sites (Table 1). The mean PD in diseased sites was 4.70 mm, which was significantly deeper than that of healthy sites (2.32 mm). The mean GI score of diseased sites was 1.5, which was significantly higher than that of healthy sites. A significant difference was observed in the BOP-positive rate between diseased and healthy sites (diseased = 40.0 vs healthy = 0.0; *P* < 0.01). Furthermore, the mean BL rate of peri-implant disease sites was 42.7%, which was approximately 2.2-fold that of healthy sites (19.7%).

### Comparison of calprotectin and NTx levels between diseased and healthy sites

**Fig. 1:**
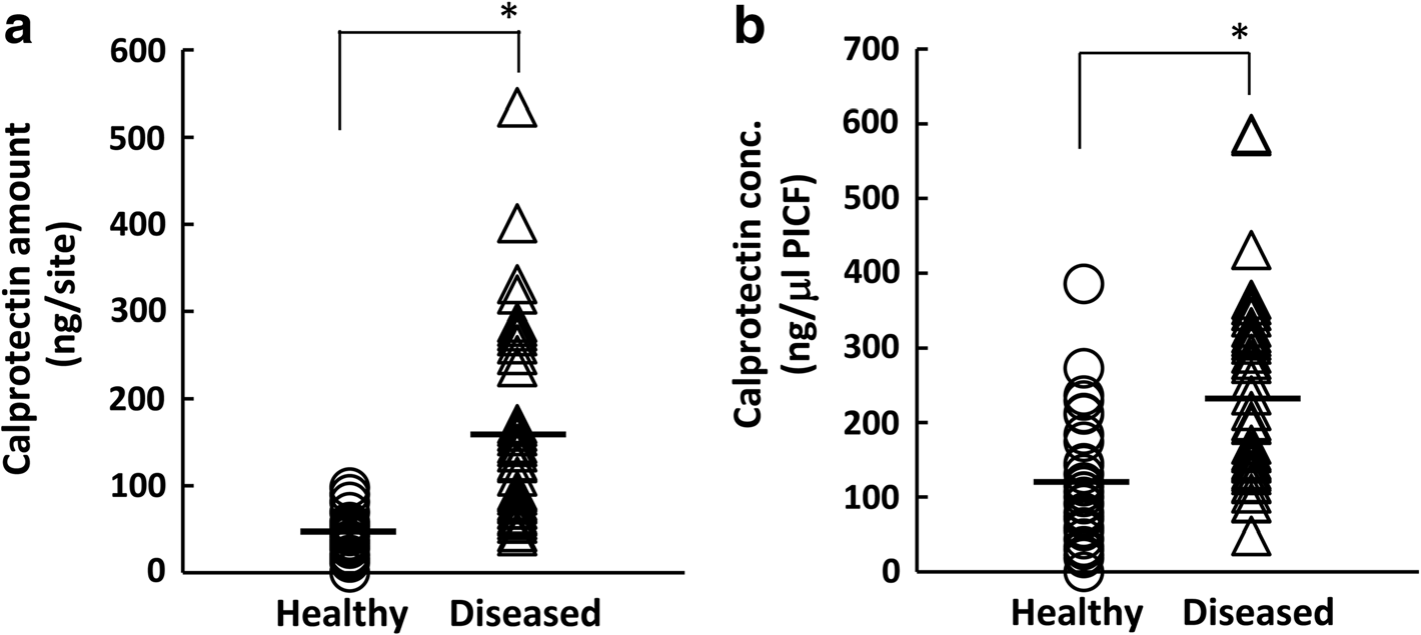
Comparison of calprotectin levels in PICF. PICF samples were collected from peri-implant disease sites (*n* = 40, diseased) and non-diseased sites (*n* = 34, healthy). Calprotectin amounts (**a**) were measured by ELISA, and its concentration (**b**) was normalized by the volume of PICF. Horizontal bars show the mean values of each group. *P < 0.01

Mean calprotectin amounts in PICF samples from diseased and healthy sites were 171.9 and 40.1 ng per site, respectively (Fig. 1a), and their mean concentrations were 231.7 and 113.2 ng/μl PICF, respectively (Fig. 1b). Calprotectin amounts and concentrations in the diseased group were significantly higher than those in the healthy group by approximately 4.3-fold and 2.1-fold, respectively (healthy vs diseased; *P* < 0.01).

**Fig. 2:**
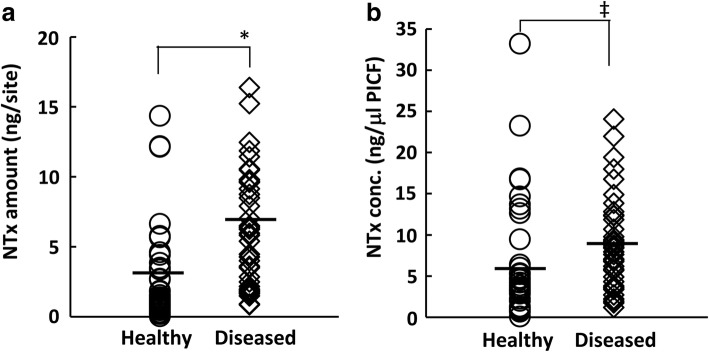
Comparison of NTx levels in PICF. NTx amounts (**a**) in PICF samples from peri-implant disease sites (*n* = 40, diseased) and non-diseased sites (*n* = 34, healthy) were measured by ELISA, and its concentration (**b**) was normalized by the volume of PICF. Horizontal bars show the mean values of each group. ^‡^*P* < 0.05, *P < 0.01

NTx amounts in PICF samples from healthy sites ranged between 0.03 and 14.34 ng per site, while those in samples from diseased sites were between 0.85 and 16.38 ng per site (Fig. 2a). Mean NTx amounts were 6.16 and 2.94 ng per site in PICF samples from the diseased and healthy groups, respectively, while the mean concentrations of NTx in the diseased and healthy groups were 9.27 and 6.62 ng/μl PICF, respectively (Fig. 2b). NTx levels in PICF samples were significantly higher from diseased sites than from healthy sites (healthy vs diseased: NTx amount *P* < 0.01, NTx concentration *P* < 0.05).

### Relationship between calprotectin amounts in PICF and clinical indicators

**Fig. 3:**
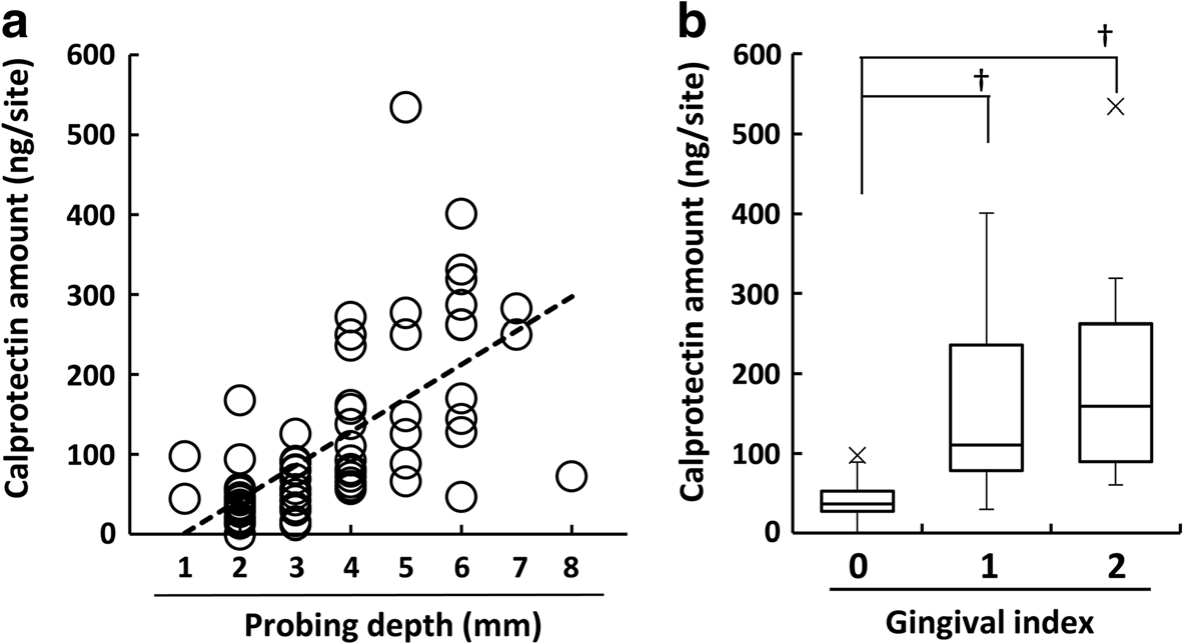
Relationship between PICF calprotectin amounts and PD or GI scores. **a** The relationship between PICF calprotectin amounts and PD was evaluated in PICF samples from peri-implant disease and healthy groups (*n* = 74, *ρ* = 0.709, *P* < 0.001). **b** Relationship between PICF calprotectin amounts and GI scores. Calprotectin amounts in PICF samples from sites with GI-0 (*n* = 34), GI-1 (*n* = 20), and GI-2 (*n* = 20) were statistically analyzed. Horizontal bars show the median of each group. ^†^*P* < 0.001

The PD range in all PICF sampling sites was 1–8 mm and calprotectin amounts ranged between 0.1 and 534.1 ng per site (Fig. 3a). A positive correlation was observed between calprotectin amounts in PICF samples and PD (*ρ* = 0.709, *P* < 0.001). The relationship between calprotectin amounts in PICF samples and GI scores was investigated (Fig. 3b). The median of calprotectin amounts in PICF samples were 36.8, 110.3, and 159.3 ng at GI-0, GI-1, and GI-2 sites, respectively. Calprotectin amounts in PICF samples from sites with GI-1 and GI-2 were significantly higher than those from sites with GI-0 (GI-0 vs GI-1 or GI-2, *P* < 0.001); however, no significant difference was noted in calprotectin amounts between GI-1 and GI-2.

### Relationship between NTx amounts in PICF and PD or BL rate

**Fig. 4:**
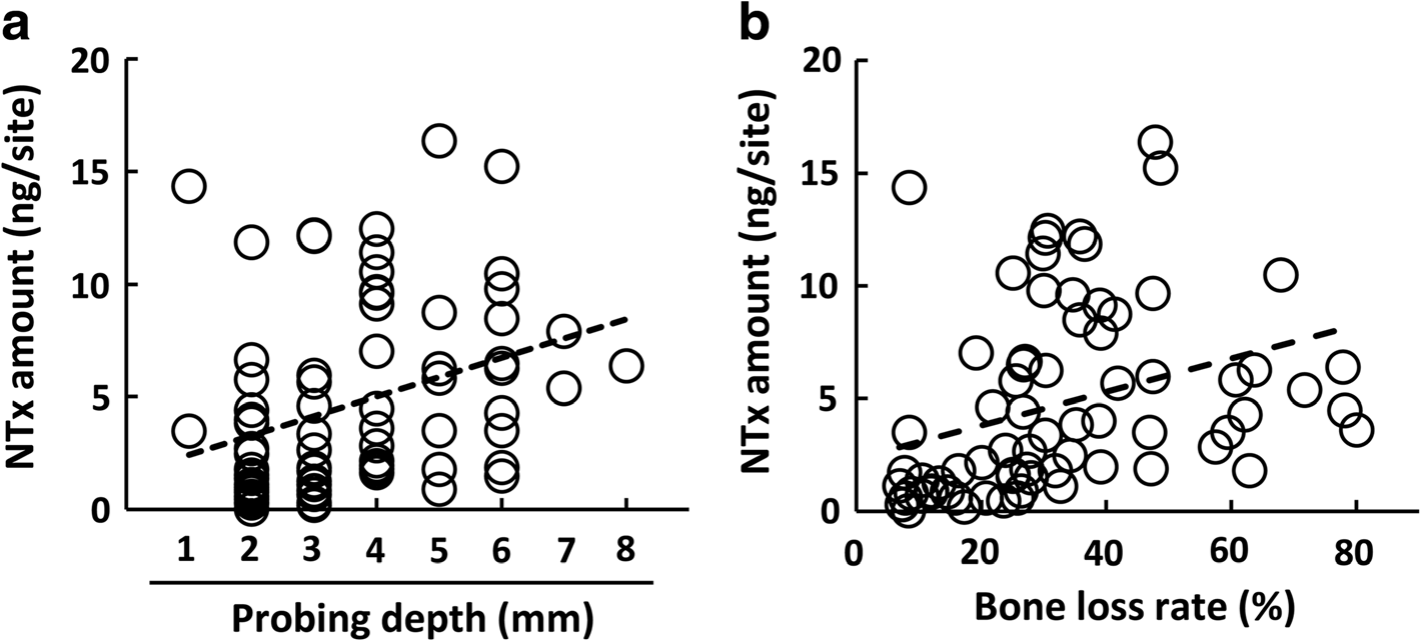
Correlation between NTx amounts and PD or BL rates. **a** The correlation between PICF NTx amounts and PD was evaluated in PICF samples from peri-implant disease and healthy groups (*n* = 74, *ρ* = 0.434, *P* < 0.001). **b** The correlation between PICF NTx amounts and BL rates (%) was evaluated in PICF samples from peri-implant disease and healthy groups (*n* = 74, *ρ* = 0.570, *P* < 0.001)

NTx amounts in PICF samples correlated with PD at PICF sampling sites (*ρ* = 0.434, *P* < 0.001, Fig. 4a). The BL rate in healthy sites ranged between 6.9 and 41.8%, while that in diseased sites was between 7.7 and 80.0% (Fig. 4b). A positive correlation was observed between NTx amounts and the BL rate (*ρ* = 0.570, *P* < 0.001).

### ROC analysis for cutoff values of calprotectin and NTx amounts in PICF

**Fig. 5:**
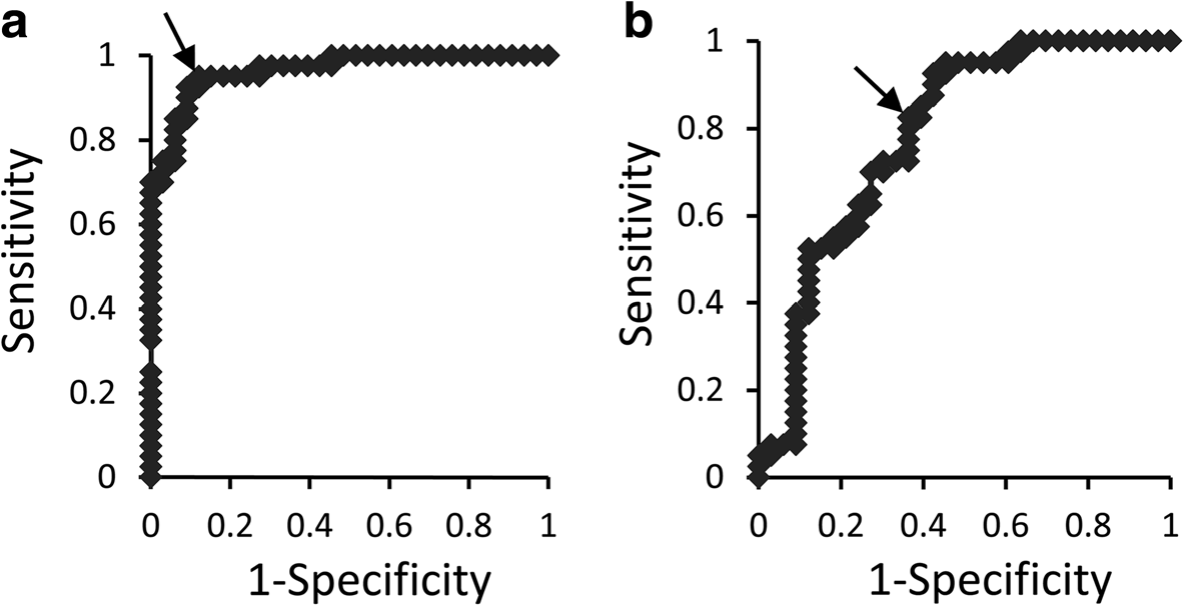
ROC analyses of PICF calprotectin and NTx to predict peri-implant diseases. PICF samples were collected from sites with and without peri-implant diseases (*n* = 74). Calprotectin (**a**) and NTx (**b**) amounts in PICF samples were subjected to ROC curve analysis. AUC values for calprotectin and NTx amounts were 0.964 (95% CI = 0.913–0.996, *P* < 0.001) and 0.784 (95% CI = 0.672–0.891, *P* < 0.001), respectively, when cutoff values were 60.4 ng/site (arrow in **a**) and 1.88 ng/site (arrow in **b**)

ROC curves for calprotectin and NTx levels in PICF were plotted in order to predict peri-implant diseases. The area under the ROC curve (AUC) for calprotectin amounts was 0.964 (95% CI = 0.913–0.996, *P* < 0.001) and the cutoff value was 60.4 ng per site, with a sensitivity of 92.5% and specificity of 90.9% (Fig. 5a). The AUC for NTx amounts was 0.784 (95% CI = 0.672–0.891, *P* < 0.001) and the cutoff value was 1.88 ng per site, with a sensitivity of 82.5% and specificity of 63.6% (Fig. 5b).

## Discussion

Diagnostic studies on peri-implant diseases using biomarkers in PICF have been performing because clinical indicators do not necessarily lead to an accurate evaluation of peri-implant diseases [5, 7, 8, 32]. Calprotectin levels were significantly higher in periodontitis GCF than in healthy GCF, and thus, calprotectin is regarded as a useful inflammatory marker for periodontal diseases [16, 17, 19]. Calprotectin amounts in PICF were measured, and its levels did not significantly change between 2 and 3 years after the functional loading of dental implants [20]. However, calprotectin levels in PICF samples from sites with and without peri-implant diseases have not yet been investigated. The present study demonstrated that calprotectin amounts and concentrations in PICF samples were significantly higher from diseased sites than from healthy sites, and a positive association was observed between calprotectin levels and clinical indicators such as PD and GI scores. This result for peri-implant diseases was similar to previous findings obtained in diagnostic studies on periodontal diseases [16, 33]. A significant difference was noted in calprotectin amounts between GI-0 group and GI-1 or GI-2 group, suggesting that PICF calprotectin indicates initial, weak inflammation in peri-implant diseases because calprotectin is mainly existed in leukocytes that more express at early stage of inflammation and acute inflammation [14, 15]. In contrast, there was a little difference of the median of calprotectin level between the GI-1 and GI-2 groups, but not significant difference, supposing that calprotectin amounts may reach to almost the maximum level at inflammation sites with GI-1 and GI-2. The ability of some biomarkers including pro-inflammatory cytokines, inflammation-related factors, and proteolytic enzymes to diagnose peri-implant diseases has been examined [5, 7, 8, 32]. IL-1β, IL-6, and PGE_2_ levels in PICF were significantly higher from peri-implantitis sites than from healthy implant sites [10, 34, 35]. However, Aboyoussef et al. [36] and Melo et al. [37] showed no significant differences in IL-1β, IL-6, and PGE_2_ levels between peri-implantitis and healthy groups. These reports indicate an opposite result, which IL-1β, IL-6, and PGE_2_ are reliable markers to detect peri-implant diseases or not. In contrast, PICF calprotectin levels showed very high sensitivity (92.5%) and specificity (90.9%) for a diagnosis of peri-implant diseases when the cutoff value was 60.4 ng per site. The sensitivity and specificity of PICF calprotectin were higher than those of AST activity, which was higher in PICF from peri-implant diseases sites than from healthy sites, with a sensitivity = 81% and specificity = 74% [12]. MMP-8 levels were previously reported to be increased in PICF from sites with peri-implantitis [11], and MMP-8 levels in PICF from peri-implant disease sites correlated with GI scores (*ρ* = 0.772, *P* < 0.001) [38]. The correlation observed between PICF calprotectin levels and GI scores in the present study (*ρ* = 0.744, *P* < 0.001, data not shown) was similar to the relationship between MMP-8 levels and GI scores.

We did not classify peri-implant diseases into peri-implant mucositis and peri-implantitis in this pilot study. Peri-implant mucositis does not show BL, whereas peri-implantitis shows BL of more than 2.5 or 3 mm on intra-oral radiographs [39, 40]. Figuero et al. [2] introduced plural diagnostic criteria for peri-implant mucositis and peri-implantitis. Rakic et al. [5] defined peri-implantitis as a PD of more than 5 mm, BOP positive, and BL of at least two threads of implant. Furthermore, Sanz et al. [41] proposed their opinion for the radiographic assessment of alveolar bone in peri-implant treatment. However, difficulties are associated with accurately measuring 2–3 mm of alveolar BL on a radiograph taken by a regular method and assessing BL levels by implant threads when implant species differ. We evaluated BL around dental implants using Schei et al.’s method [30], which has been used to evaluate BL rate in periodontal diseases. The mean BL rate was significantly higher at peri-implant disease site than at healthy sites without inflammation and deep PD. Therefore, we did not distinguish peri-implant mucositis and peri-implantitis that were diagnosed by measuring bone level on radiograph in the present pilot study. Biomarkers for BL may be more accurate than clinical BL indicators because PICF NTx amounts were found to correlate with BL rates determined by Schei et al.’s method (*ρ* = 0.570, *P* < 0.001). Biomarkers for bone metabolism in PICF and clinical, radiological assessment of bone level may accurately diagnose peri-implant mucositis and peri-implantitis.

Bone-related proteins including ICTP, osteocalcin (OCN), and RANKL have been studied as BL biomarkers in peri-implantitis. ICTP, a cross-linked C-telopeptide of type I collagen, is a marker for bone degradation, and its levels in PICF were significantly higher from peri-implantitis sites than from healthy sites [9, 42]. However, Tümer et al. [13] did not detect a significant difference in PICF ICTP levels between peri-implantitis and healthy sites. RANKL is a main mediator of osteoclast formation and associated with bone resorption [43]. Soluble RANKL (sRANKL) concentrations in PICF were significantly higher from peri-implantitis sites than from healthy implant sites (*P* < 0.01), and its levels correlated with clinical indicators such as PD (*ρ* = 0.309, *P* = 0.034) and BOP (*ρ* = 0.327, *P* = 0.024) [44]. In the present study, NTx amounts and concentrations showed similar significant differences to sRANKL between the peri-implant disease and healthy groups (amount: *P* < 0.01, concentration: *P* < 0.05), and a stronger correlation was observed between NTx amounts and PD (*ρ* = 0.434, *P* < 0.001). In contrast, Arikan et al. [9] showed that sRANKL concentrations in PICF were significantly higher in healthy groups, while Sarlati et al. [45] reported no significant difference in PICF sRANKL concentrations among healthy, peri-implant mucositis, and peri-implantitis groups. OCN is a major non-collagenous protein in bone and is associated with bone metabolism [46]. The mean OCN concentration in PICF from peri-implantitis sites was approximately 1.5-fold that of healthy groups [13], and this finding was similar to the result for NTx in PICF. Although OCN levels in PICF samples were significantly higher from peri-implant mucositis sites without BL than from healthy sites, OCN levels in PICF from peri-implantitis with BL was not significantly different from those in PICF from healthy and peri-implant mucositis sites [47]. These conflicting findings do not necessarily suggest that ICTP, sRANKL, and OCN are reliable biomarkers for alveolar BL. Few studies showed a relationship between the PICF levels of bone-related markers and those of clinical indicators for alveolar BL. NTx levels in GCF samples were significantly higher from periodontitis sites than from healthy sites [28]; however, the relationship between NTx levels in PICF or GCF and BL levels has not yet been investigated. NTx in PICF may be a reliable biomarker for evaluating BL in peri-implantitis because PICF NTx levels correlated with the BL rate as well as PD and had high sensitivity and specificity for predicting peri-implant diseases.

Treatments for peri-implant diseases are selected by CIST [6], in which clinical indicators including PD, BOP, implant mobility, and BL on radiographs are used to diagnose peri-implant diseases. However, these clinical indicators are not considered to be sufficiently accurate or objective for the diagnosis of peri-implant diseases. Biomarkers in PICF contribute to the diagnosis of peri-implant diseases by clinical indicators and may provide a reliable diagnosis of onset, progression, and prognosis of disease as well as the selection of treatments. This pilot study suggests that calprotectin and NTx in PICF may be useful biomarkers for the diagnosis of peri-implant diseases, and future study using a large number of PICF samples will support the results obtained herein.

## Conclusions

Calprotectin and NTx in PICF are markers of inflammation and bone resorption in peri-implant tissues and may be useful diagnostic markers for peri-implant diseases.

## Abbreviations

AST: Aspartate aminotransferase
BL: Bone loss
BOP: Bleeding on probing
CIST: Cumulative interceptive supportive therapy
GCF: Gingival crevicular fluid
GI: Gingival index
ICTP: Cross-linked C-telopeptide of type I collagen
IL-1β: Interleukin-1β
MMP-8: Matrix metalloproteinase-8
NF-κB: Nuclear factor-κB
NTx: Cross-linked N-telopeptide of type I collagen
OCN: Osteocalcin
PD: Probing depth
PICF: Peri-implant crevicular fluid
RANKL: Receptor activator of NF-κB ligand
sRNAK: Soluble RANKL
TMB: 3,3′,5,5′-Tetramethylbenzidine
TNF-α: Tumor necrosis factor-α
